# A model-free and distribution-free multi-omics integration approach for detecting novel lung adenocarcinoma genes

**DOI:** 10.1038/s41598-023-45813-w

**Published:** 2024-08-03

**Authors:** Shaofei Zhao, Caleb Qi, Geran Zhao, Yangsheng Wang, Guifang Fu

**Affiliations:** Binghamton University, Department of Mathematics and Statistics, Binghamton, NY 13902 USA

**Keywords:** Statistics, Cancer genomics, Gene expression, Genomics, Scientific data

## Abstract

Detection of important genes affecting lung adenocarcinoma (LUAD) is critical to finding effective therapeutic targets for this highly lethal cancer. However, many existing approaches have focused on single outcomes or phenotypic associations, which may not be as thorough as investigating molecular transcript levels within cells. In this article, we apply a novel multivariate rank-distance correlation-based gene selection procedure (MrDcGene) to LUAD multi-omics data downloaded from The Cancer Genome Atlas (TCGA). MrDcGene provides additional opportunities for detecting novel susceptibility genes as it leverages information from multiple platforms, while efficiently handling challenges such as high dimensionality, low signal-to-noise ratio, unknown distributions, and non-linear structures, etc. Notably, the MrDcGene method is able to detect two different scenarios, i.e., strong association strength with a few gene expressions and weak association strength with several gene expressions. After thoroughly exploring the association between gene expression (GE) and multiple other platforms, including reverse phase protein array (RPPA), miRNA, copy number variation (CNV) and DNA methylation (ME), we detect several novel genes that may play an important role in LUAD (*ZNF133, CCDC159, YWHAZ, HNRNPR. ITPR2, PTHLH*, and *WIPI2*). In addition, we quantitatively validate several other susceptibility genes that were reported in the literature using different methods and studies. The accuracy of the MrDcGene approach is theoretically assured and empirically demonstrated by the simulation studies.

## Introduction

As one of the most frequently diagnosed cancers in the world, lung cancer ranks first in mortality among all cancer types in both men and women. In 2020, there were 2,206,771 new cases (11.4% of all cancer types) and 1,796,114 new deaths (18.0% of all cancer types) were associated with lung cancer^1^. Lung adenocarcinoma (LUAD) is the most common histologic type of lung cancer. In addition to environmental factors such as smoke^2,3^, a large number of never-smokers have LUAD, which indicates the influence of genetic factors on LUAD. For example, epidermal growth factor receptor (*EGFR*) is one of the most represented mutations in LUAD patients, especially in Asians (51.4% of Asian patients have such mutation), and thus can be a sensitive therapeutic target for tyrosine kinase inhibitors (TKI)^4^. However, a large proportion of LUAD patients still lack effective therapeutic targets, and therefore, more biomarkers are needed for LUAD^5^.

A single outcome, such as survival, binary disease status, or a clinical endpoint, may not sufficiently capture all the characteristics of cancer progression. Furthermore, exploration of phenotypic association alone may not be as thorough as investigating molecular transcript levels within cells. Therefore, leveraging information from multiple platforms holds the potential for gaining additional advantages and presenting more opportunities to uncover new findings compared to single-omics analysis^5–8^. However, effectively handling challenges such as high dimensionality, low signal-to-noise ratio (SNR), unknown distributions, and messy structures generated from multiple different platforms calls for new data analysis strategies^6^.

We download the multi-omics LUAD data from the Cancer Genome Atlas (TCGA) (https://www.cancer.gov/about-nci/organization/ccg/research/structural-genomics/tcga), which is a significant cancer genomics program funded by the National Cancer Institute (NCI) and the National Human Genome Research Institute. It plays a vital role in enhancing our research about diagnose, treat, and prevent cancer. These multi-omics datasets contain Reverse Phase Protein Array (RPPA), miRNA, copy number variation (CNV), DNA methylation (ME), and gene expression (GE).

Existing approaches analyzed multi-omics data through a joint model by stacking all variables from multiple platforms as a long vector. Then they modeled the relationship between a clinical outcome (like survival rate) and this long vector containing all platforms by various dimension reduction approaches^6,9^. For example, Zhao et al. (2015) applied principal component analysis, partial least squares, and LASSO to reduce the dimension of each platform independently and then fitted a Cox survival model onto reduced features that were combined from multiple platforms^10^. Takahashi et al. (2020) utilized an auto-encoder machine learning skill to reduce the dimension of each platform to 100 variables, and then further selected clinically meaningful features from the compressed $100\times 6$ features using a Cox model^7^. Other studies considered both the joint and individual relationships simultaneously. For example, Lock and Dunson (2013) proposed a Bayesian consensus clustering (BCC) method to perform classifications based on both an overall structure and individual structures of each of the platforms^11^. Lock et al. (2013) proposed a general decomposition framework entitled joint and individual variation explained (JIVE) to extract low rank approximation for joint and individual latent components^12^. Gaynanova and Li (2019) improved JIVE by adding a structural sparsity pattern on the factorization term to further incorporate partially shared structures^13^. However, the existing methods have some limitations that leave room for alternative strategies. For example, they assumed linear models and/or certain prior distributions; eigenvalue decomposition may be computationally expensive or unstable for high-dimensional data; and pleiotropy may be ignored in cases where one gene affects multiple expressions but its signal is weak in each expression.

In this article, we apply a new multivariate rank distance correlation-based gene selection procedure (MrDcGene) to LUAD multi-omics data^14^. This approach represents the latest advancement in sure independence screening methods. In 2008, Fan and Lv^15^ initially introduced the sure screening concept, meaning that as the number of observations $n\rightarrow \infty$, all true predictors can be selected with a probability approaching 1. They employed the Pearson correlation as the marginal dependence score between the response and each of the predictor, ranked them from highest to lowest, and select the top predictors. This approach, known as Sure Independence Screening (SIS), was shown to possess the sure screening property, and is suitable for ultrahigh dimensional predictor spaces where the number of predictors *p* can be quite large ($\log (p) = O(n^\xi )$ for some $\xi >0$). SIS gained a lot of attention, leading to the development of several other sure screening approaches. One such approach, introduced by Li et al.^16^ in 2012, was based on distance correlation (DC-SIS). DC-SIS relaxed the normality assumption inherent in SIS, and extended its applicability to mutli-dimensional responses. However, it still required the data to satisfy sub-exponential tail bounds, which might not be met in the presence of heavy-tailed data. MrDcGene represents a further relaxation of this requirement, making it particularly well-suited for analyzing multi-omics data. Its contributions to the gene selection literature are characterized by several attractive properties. (1) It is entirely model-free, mitigating concerns related to model misspecification, and is applicable in both linear and nonlinear relationships. (2) It is distribution-free, it does not assume data to be normally distributed or to follow any particular distribution. After performing exploratory data analysis, we noticed that nonlinearity, non-normality and skewness are common in this messy TCGA-LUAD data. (3) It retains the sure screening property, ensuring that it will not overlook a true gene when the number of observations is sufficiently large. (4) It is adaptable to multiple platforms without incurring additional costs, this feature is particularly advantageous for multi-omics data analysis. In addition, correlation of multiple platforms for the same matched gene can be incorporated.

We conduct an in-depth investigation through four studies: association of GE with RPPA-Protein; association of GE with miRNA; association of five GEs with integrated CNV and ME; and association of all matched GEs with integrated CNV and ME. We not only identify genes that have extremely strong associations with the expressions of a small number of genes, but also recognize others that are associated with expressions of multiple genes while each having weak strength. These investigations also indirectly suggest pleiotropic and epistatic effects of multiple genes that may regulate LUAD. We identify several important genes as potential biomarkers for LUAD cancer, some confirm those reported in the literature and other findings are novel. The accuracy of this real data analysis is theoretically supported by the proof presented by Zhao and Fu^14^ and empirically guaranteed by the simulation study in this article with weak signal-to-noise ratio and high dimensionality.

## Results

### Synthetic data simulation

**Study 1**: Assessing the robustness of MrDcGene in detecting simple nonlinear relationships.

To begin, we demonstrate the robustness of MrDcGene in detecting simple nonlinear relationships. Since the SIS is based on Pearson correlation and it can only handle one-dimensional data. We generate simulation data as follows.

Set the number of observations to be $n = 200$, the number of predictor vectors to be $p = 10,000$, and the dimension of response variable to be $q = 1$. To mimic real-world data (especially human genetics data), we generate $\varvec{X}_{n\times p}$ from multivariate $t_1$ distribution (*t* distribution with degrees of freedom 1), with zero mean and AR(1) covariance matrix ${\varvec{\Sigma }}_{p\times p} = [\sigma _{ij}]_{p\times p}$, where $\sigma _{ij} = 0.8^{|i-j|}$ for $i,j = 1,2,\dots ,p$. Next, we construct the response $\varvec{y}_{n\times 1}$ as

$$ \varvec{y}= \beta _1 \varvec{X}_{1} + \beta _2 \varvec{X}_{6} + \beta _3 \varvec{X}_{12}^2 + \beta _4 \varvec{X}_{22} + \varvec{\epsilon }, $$

where $\varvec{\epsilon }_{n\times 1}$ also follows $t_1$ distribution, and $\beta _{1,2,3,4}\sim Uniform (2, 5)$.

We repeat the simulation 100 times and following Li et al.^16^, we utilize three criteria to assess the performance of SIS, DC-SIS and MrDcGene:

- $\mathcal {S}$: This stands for the minimum model size required to include all true predictors. We report the 25th, 50th, and 95th percentiles of $\mathcal {S}$ across 100 replicates. The smaller the $\mathcal {S}$, the better the performance.
- $\mathcal {P}_s$: This represents the individual success rate of selecting a single true predictor within a predetermined cutoff across the 100 replicates. The larger the individual $\mathcal {P}_s$, the better the performance.
- $\mathcal {P}_a$: This measures the overall success rate of selecting all true predictors simultaneously within the predetermined cutoff across the 100 replicates. The larger the $\mathcal {P}_a$, the better the performance.

Consistent with the recommendation of Li et al.^16^ we set the predetermined cutoff as $[n/\log (n)]$, when $n = 200$, the pre-determined cutoffs are $s_1 = [n/\log (n)] = 37$, $s_2 = 2s_1$ and $s_3 = 3s_1$. To provide an intuitive assessment of performance, we also create a boxplot for the rank of each true predictor. In this context, a smaller rank indicates that the predictor is more important. Idealy, the true predictors should be ranked at the top.

**Figure 1:**
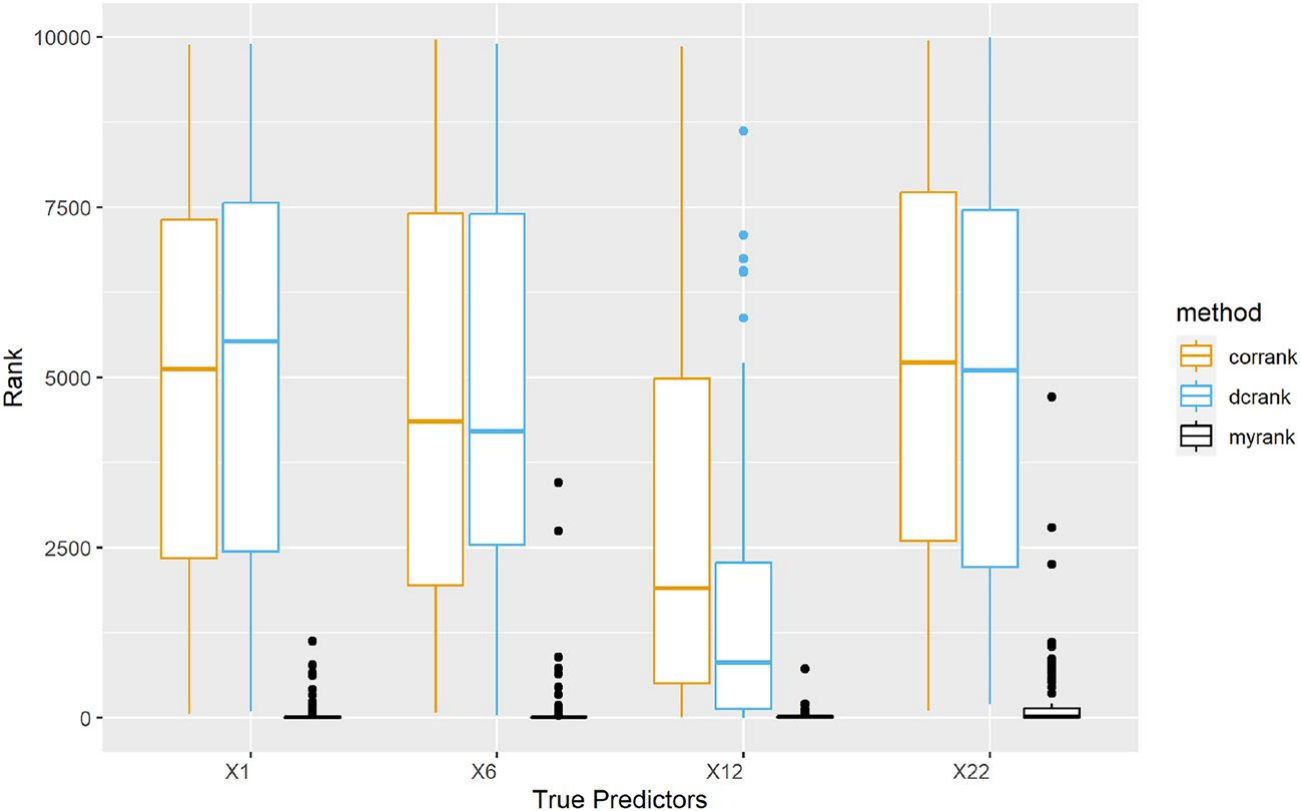
Rank of true predictors in Study 1, the smaller the rank, the better the performance. Orange: SIS. Blue: DC-SIS. Black: MrDcGene.

The results of all three methods are summarized in Fig. 1 and Table 1. A clear observation from Fig. 1 is that MrDcGene consistently outperforms the other two methods across all 4 true predictors, consistently ranking them at the top. In Table 1, we note that the 95th percentile of the minimum model sizes $\mathcal {S}$, required to include all true predictors for both SIS and DC-SIS, exceeds 9800. This suggests that these methods struggle to distinguish true predictors from noise effectively. In contrast, MrDcGene exhibits a 50th percentile of $\mathcal {S}$ at only 100, and a 95th percentile at 1100. This superior performance of MrDcGene when the data is from $t_1$ distribution, is noteworthy. It consistently ranks all 4 true predictors at the top, and demonstrates robustness to nonlinear relationships, as exemplified by $\varvec{X}_{12}^2$. Since SIS is based on Pearson correlation, it has difficulty in detecting $\varvec{X}_{12}$, resulting in an average ranking at around 2000. DC-SIS, which is robust to nonlinear relationships, performs better on $\varvec{X}_{12}$, but still falls short of MrDcGene’s performance. As the square term dominating the model, all three methods rank $\varvec{X}_{12}$ prior to other 3 predictors, but SIS and DC-SIS cannot distinguish other 3 true predictors from noise, with an average ranking of around 5,000.

**Figure 2:**
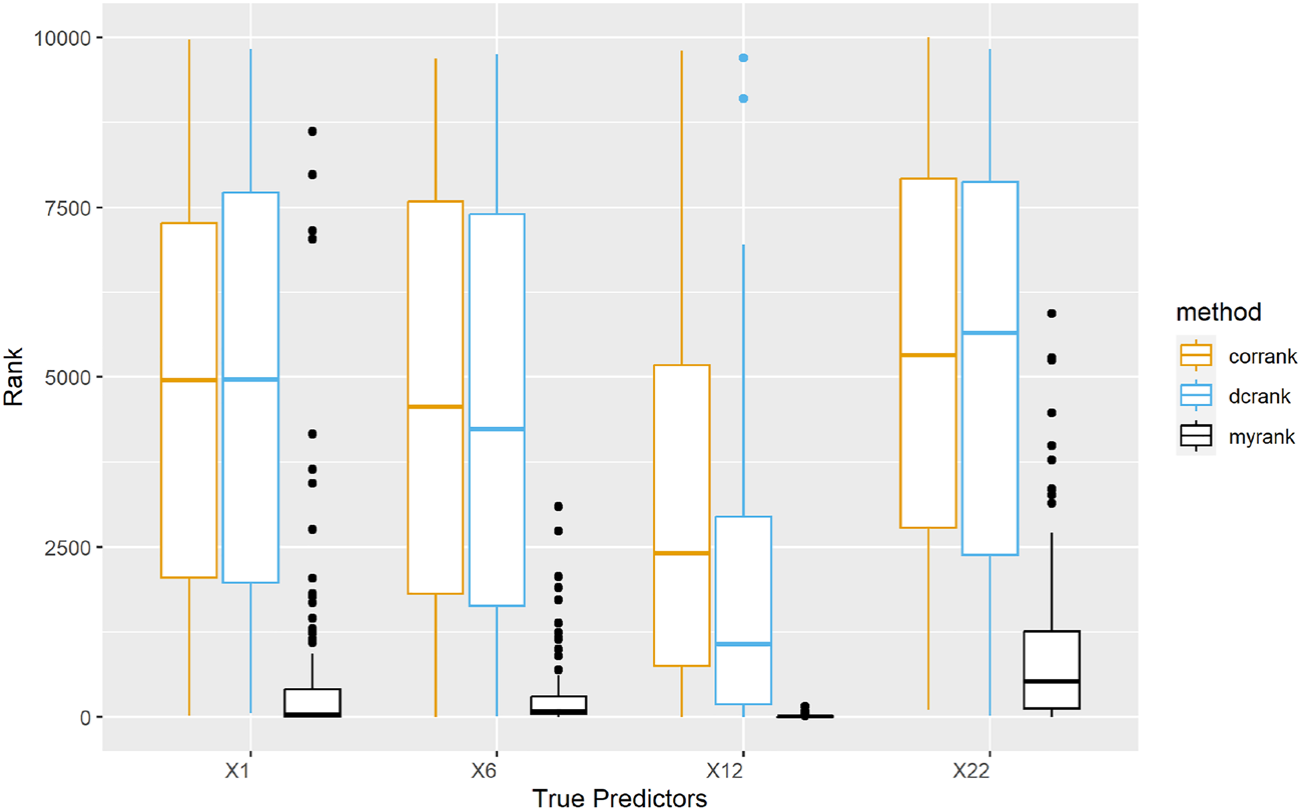
Rank of true predictors in Study 2, the smaller the rank, the better the performance. Orange: SIS. Blue: DC-SIS. Black: MrDcGene.

**Table 1:** Performance of the three approaches for Study 1.

Method	$\mathcal {S}$		Selection size	$\mathcal {P}_{\varvec{X}_1}$	$\mathcal {P}_{\varvec{X}_6}$	$\mathcal {P}_{\varvec{X}_{12}}$	$\mathcal {P}_{\varvec{X}_{22}}$	$\mathcal {P}_a$
SIS	25%	6777.25	$s_1$	0	0	0.08	0	0
	50%	8118	$s_2$	0.01	0.01	0.1	0	0
	95%	9887.05	$s_3$	0.01	0.01	0.12	0.01	0
DC-SIS	25%	6558.75	$s_1$	0	0	0.13	0	0
	50%	7935	$s_2$	0	0.01	0.2	0	0
	95%	9805.25	$s_3$	0.01	0.01	0.22	0	0
MrDcGene	25%	28.75	$s_1$	0.82	0.84	0.86	0.61	0.31
	50%	99.5	$s_2$	0.88	0.89	0.93	0.68	0.44
	95%	1180.3	$s_3$	0.89	0.9	0.95	0.72	0.52

The minimum selection size $\mathcal {S}$, the individual success rate $\mathcal {P}_s$, and the overall success rate $\mathcal {P}_a$ are demonstrated. The predetermined cutoffs for $\mathcal {P}_s$ and $\mathcal {P}_a$ are $s_1 = [n/\log (n)]=37$, $s_2 = 2s_1$ and $s_3 = 3s_1$.

**Study 2**: Assessing the robustness of MrDcGene in detecting complex nonlinear relationships.

To further demonstrate the robustness of MrDcGene, we conduct a simulation with more complex relationships. As in Study 1, we set the number of observations to be $n = 200$, the number of predictor vectors to be $p = 10,000$, and the dimension of response variable to be $q = 1$. We generate $\varvec{X}_{n\times p}$ the same way as in Study 1, and construct the response $\varvec{y}_{n\times 1}$ as

$$ \varvec{y}= \beta _1 \varvec{X}_1 + \beta _2 \log (\varvec{X}_6) + \beta _3 \varvec{X}_{12}^2 + \beta _4 |\varvec{X}_{22}|+ \varvec{\epsilon }, $$

where $\varvec{\epsilon }\sim t_1$ and $\beta _{1,2,3,4}\sim Uniform (2,5)$. Now the response is connected to the predictors via more complex and nonlinear relationships.

The results are presented in Fig. 2 and Table 2. With more complex relationships, SIS and DC-SIS exhibit similar performance to that observed in Study 1, with the 95th percentile of $\mathcal {S}$ for both methods exceeding 9,700. Given the total predictor size $p = 10,000$, there is not much room for deterioration. MrDcGene, while performing somewhat worse than in Study 1, still outperforms SIS and DC-SIS across all 4 true predictors. In Fig. 2, MrDcGene consistently ranks all 4 predictors at the top, with a little difficulty in selecting $\varvec{X}_{22}$. Like Study 1, $\varvec{X}_{12}^2$ remains the dominant predictor, consistently ranking first compared to the other 3 true predictors, and the success rate of selecting $\varvec{X}_{12}$ given a fixed model size is always the highest in each method. However, since SIS is designed for linear relationships, it still ranks $\varvec{X}_{12}$ at an average of around 2,500. DC-SIS performs slightly better, with an average ranking of $\varvec{X}_{12}$ at around 1,200. MrDcGene excels in this regard, consistently ranking $\varvec{X}_{12}$ at the top, surpassing the other two methods. Regarding the log ($\varvec{X}_6$) and absolute value ($\varvec{X}_{22}$) relationships, MrDcGene continues to perform well, while SIS and DC-SIS rank the true predictors at around 5,000 on average.

**Table 2:** Performance of the three approaches for Study 2.

Method	$\mathcal {S}$		Selection size	$\mathcal {P}_{\varvec{X}_1}$	$\mathcal {P}_{\varvec{X}_6}$	$\mathcal {P}_{\varvec{X}_{12}}$	$\mathcal {P}_{\varvec{X}_{22}}$	$\mathcal {P}_a$
SIS	25%	6872.75	$s_1$	0.01	0.02	0.08	0	0
	50%	8030.5	$s_2$	0.01	0.04	0.09	0	0
	95%	9802.3	$s_3$	0.01	0.04	0.1	0.01	0
DC-SIS	25%	6584	$s_1$	0	0.02	0.16	0.01	0
	50%	8111	$s_2$	0.02	0.03	0.21	0.01	0
	95%	9761.15	$s_3$	0.02	0.05	0.22	0.01	0
MrDcGene	25%	327.75	$s_1$	0.53	0.26	0.92	0.1	0
	50%	1071.5	$s_2$	0.57	0.49	0.97	0.15	0.02
	95%	5313	$s_3$	0.62	0.56	0.98	0.21	0.04

The minimum selection size $\mathcal {S}$, the individual success rate $\mathcal {P}_s$, and the overall success rate $\mathcal {P}_a$ are demonstrated. The predetermined cutoffs for $\mathcal {P}_s$ and $\mathcal {P}_a$ are $s_1 = [n/\log (n)]=37$, $s_2 = 2s_1$ and $s_3 = 3s_1$.

**Study 3**: Assessing MrDcGene’s performance in emulating real-world data.

To closely emulate multi-omics data and evaluate the performance of the MrDcGene approach under high dimensions and low signal-to-noise ratios across multiple platforms, we conduct the following simulation and compare the performance of MrDcGene with DC-SIS. Since SIS can only handle data with a single-dimensional response, it is not considered in studies involving multi-dimensional responses.

We set the number of observations to be $n = 200$, the number of predictor vectors to be $p = 10,000$, and the dimension of response vectors to be $q = 10,000$. Furthermore, each predictor is designed to be a 3-dimensional vector to mimic three different platforms in the multi-omics data. Specifically, we generate each of the three platforms as: $\varvec{U}= [U_1,U_2,\dots ,U_p]$ from a multivariate *t* distribution with degrees of freedom 2, $\varvec{V}= [V_1,V_2,\dots ,V_p]$ from a multivariate *t* distribution with degrees of freedom 1, and $\varvec{W}= [W_1,W_2,\dots ,W_p]$ from a multivariate *t* distribution with degrees of freedom 3, and we set the covariance matrix of $\varvec{U}, \varvec{V}$, and $\varvec{W}$ as ${\varvec{\Sigma }}_{p\times p} = [\sigma _{ij}]_{p\times p}$, where $\sigma _{ij} = 0.8^{|i-j|}$ for $i,j = 1,2,\dots ,p$. We can view each of $\varvec{U}$, $\varvec{V}$, $\varvec{W}$ as the data from one single platform, for example, $\varvec{U}$ represents the data from copy number variation, $\varvec{V}$ represents the data from DNA methylation, etc. and our predictor array $\varvec{X}= [\textbf{X}_1,\textbf{X}_2,\dots ,\textbf{X}_p]$ will be a stack of all platforms (thus multi-omics), where each predictor $\textbf{X}_j = [U_j, V_j, W_j]$, $\forall j = 1,2,\dots , p$, is further a 3-dimensional vector for each subject. Therefore, $\varvec{X}$ is a $200\times 10,000\times 3$ array or a tensor of order 3. To mimic the sparsity structure in both predictors and responses as the real data has, we set the first 12 dimensions in $\varvec{Y}$ to be the truth and the remaining 9, 988 dimensions to be the noise; we also set only one platform, not all three platforms of $\textbf{X}_2$ and $\textbf{X}_4$ to be the truth and the remaining 9, 998 predictors to be the noise. Then we connect the first 12 response dimensions with the true predictors as

$$ \varvec{Y}_k = \beta _1 \varvec{X}_{2,id_1} +\beta _2 \varvec{X}_{4,id_2} + \varvec{\epsilon }, \forall k=1,\dots , 12, $$

where $\varvec{\epsilon }\sim t_1$, and $\beta _{1,2}\sim Uniform (4,5)$. We randomly select a platform from $\varvec{U}$, $\varvec{V}$ and $\varvec{W}$ by the values of $id_1$ and $id_2$ that are randomly sampled from {1,2,3}. This better reflects the true situation, as the expression of different genes may be associated with different combinations of platforms. The remaining response dimensions $\varvec{Y}_{13}, \dots , \varvec{Y}_{10,000}$ are generated from $t_1$ distribution with degrees of freedom 1 without connecting to any predictors.

We first attempted to fit the entire 10, 000-dimensional response as a whole unit, but neither MrDcGene nor DC-SIS gives satisfactory results. Although both approaches are feasible for multi-dimensional response, the useful signal in response is too low in this simulation (signal to noise ratio is $12/(10,000-12)\approx 1.2\%$). The purpose of gene selection is to determine which genes have important associations with the response. However, if the response target itself contains a large amount of impurities, finding genes related to it is similar to looking for a needle in a haystack. In order to solve the issue, we utilize a grouping procedure, i.e., divide $\varvec{Y}$ into 2000 subgroups with each subgroup including only 5 dimensions. We then calculate the marginal dependence score between each subgroup and each $\varvec{X}_j$, by doing this we get $2000\times p$ scores in total. Notice that all $\varvec{Y}_k$’s in the first two subgroups are truly connected with $\varvec{X}_2$ and $\varvec{X}_4$; only $\varvec{Y}_{11}$ and $\varvec{Y}_{12}$ in the 3rd subgroup are connected with $\varvec{X}_2$ and $\varvec{X}_4$; and all the $\varvec{Y}_j, j=13,\ldots , p$ in the remaining subgroups are noises. Therefore, the true predictors should be located at the 2nd, 4th, 10, 002nd, 10, 004th, 20, 002nd and 20, 004th in the $2,000\times p$ scores.

The results of these two approaches are summarized in Table 3. The MrDcGene effectively detects $\varvec{X}_2$ and $\varvec{X}_4$ from $2,000\times 10,000$ scores with a fairly small selection size ($<20$) and it successfully chooses all active predictors with a simultaneous success rate of 1 at a small threshold $s_1 = 37$. On the contrary, the success rate of DC-SIS is close to zero even when threshold is three times higher, and its selection size is extremely high ($>10^4$). In this simulation, the MrDcGene performs favorably in handling multi-dimensional data when both response and predictors are in high dimension ($p=10,000$ and $q=10,000$) with weak SNR after incorporating the grouping procedure.

**Table 3:** Performances of the two approaches for Study 3.

Method	$\mathcal {S}$		Selection size	$\mathcal {P}_{\varvec{X}_2}$	$\mathcal {P}_{\varvec{X}_4}$	$\mathcal {P}_{\varvec{X}_2(\varvec{X}_{10002})}$	$\mathcal {P}_{\varvec{X}_4(\varvec{X}_{10004})}$	$\mathcal {P}_{\varvec{X}_2(\varvec{X}_{20002})}$	$\mathcal {P}_{\varvec{X}_4(\varvec{X}_{20004})}$	$\mathcal {P}_a$
DC-SIS	25%	21455.5	$s_1$	0.04	0.04	0.02	0.06	0.04	0.06	0.02
	50%	95751	$s_2$	0.08	0.1	0.06	0.08	0.06	0.08	0.02
	95%	14106407.4	$s_3$	0.08	0.12	0.06	0.1	0.06	0.08	0.04
MrDcGene	25%	11	$s_1$	1	1	1	1	1	1	1
	50%	12	$s_2$	1	1	1	1	1	1	1
	95%	16	$s_3$	1	1	1	1	1	1	1

The minimum selection size $\mathcal {S}$, the individual success rate $\mathcal {P}_s$, and the overall success rate $\mathcal {P}_a$ are demonstrated. The pre-determined cutoffs for $\mathcal {P}_s$ and $\mathcal {P}_a$ are $s_1 = [n/\log (n)]=37$, $s_2 = 2s_1$ and $s_3 = 3s_1$.

**Study 4**: Evaluating MrDcGene’s performance across different dimensions of response.

In Study 3 we observe that neither DC-SIS nor MrDcGene gives satisfactory results when using the entire 10,000-dimensional response at once, and we assume this is primarily due to the low signal-to-noise ratio (SNR) of the response. To address this, We divide the response $\varvec{Y}$ into 2000 subgroups, each containing only 5 dimensions. In this study, we want to further investigate the relationship between SNR and performance. Furthermore, to test if MrDcGene remains robust against nonlinear relationships in multi-dimensional settings, we modify the setup from Study 3 as follows: the construction of $\varvec{X}$ keeps the same. For $\varvec{Y}$, we vary the dimensionality to be $q = 2, 3, 4, 5, 6, 7, 8, 9, 10, 20, 30, 40 ,50, 60 ,70, 80$ to simulate different SNRs. Only the first dimension of $\varvec{Y}$ is connected with $\varvec{X}$ by

$$ \varvec{Y}_1 = \beta _1 \varvec{X}_{2,id_1} + \beta _2 \varvec{X}_{4,id_2} + \beta _3 \varvec{X}_{101,id_3} + \beta _4 \varvec{X}_{102,id_4}^2 + \varvec{\epsilon }, $$

where $\varvec{\epsilon }\sim t_1$, $\beta _{1,2,3,4}\sim Uniform(1,2)$, and the platform index $id_{1,2,3,4}$ are randomly sampled from {1,2,3}. The remaining dimensions of $\varvec{Y}_k$, $k = 2, 3, \dots , q$ will again be generated from $t_1$ distribution without connecting to any predictors.

**Figure 3:**
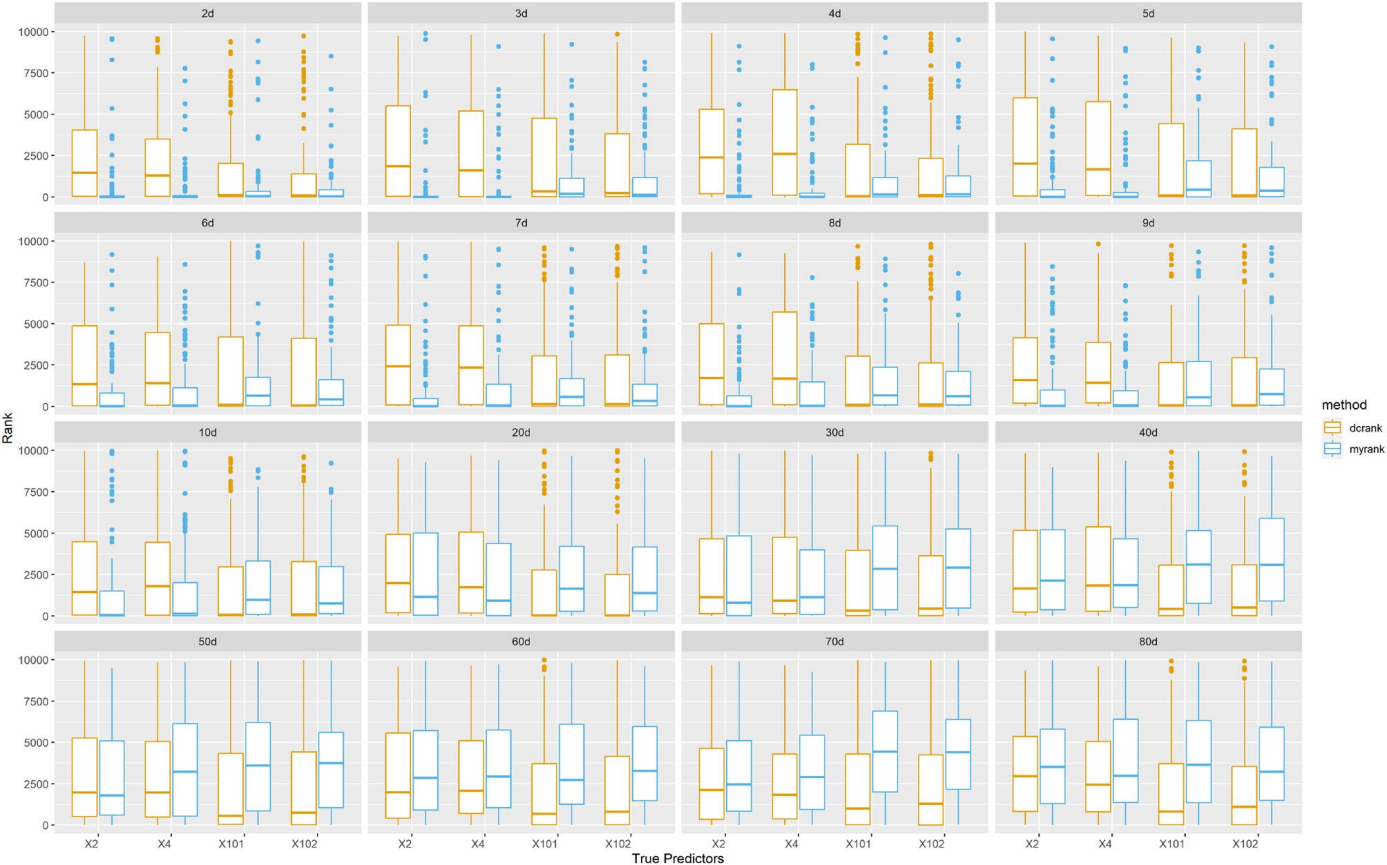
Rank of true predictors in Study 4 with different response dimensions. Orange: DC-SIS. Blue: MrDcGene.

The results are depicted in Fig. 3. We can see that as *q* increases (and SNR decreases), both methods tend to exhibit deteriorating performance. This observation aligns with our assumption that as the response becomes noisier, it becomes increasingly challenging to detect the true predictors. Specifically, when the dimension of response $q\le 8$, MrDcGene outperforms DC-SIS, with smaller ranks for all 4 true predictors compared to DC-SIS. However, when $q\ge 9$, MrDcGene starts to struggle in detecting $\varvec{X}_{101}$ and $\varvec{X}_{102}$. As the dimension further increases, MrDcGene is no longer able to distinguish the true predictors from the noise. DC-SIS faces difficulties from the beginning, and fails to distinguish the true predictors from the noise in this setting. Given these findings and to err on the side of caution in real data analyses, we choose to divide the response into 5-dimensional subgroups, as was done in Study 3.

**Study 5**: Examining MrDcGene’s computational time.

In this study, our aim is to assess the computational time required for all three methods. To get a better understanding of how dimensions affect computational time, we vary the number of observation *n* to be 200, 400 and 600, the number of predictor *p* to be 1000, 2000 and 5000, and the dimension of response *q* to be 1, 3 and 5. Notice that the SIS cannot handle multivariate responses, so the computational time for SIS is marked as “NA” in scenarios where $q>1$. For our simulations, the predictor $\varvec{X}_{n\times p}$ and the response $\varvec{Y}_{n\times q}$ are generated from standard normal distribution. We measure the excution time for each method using the Python “timeit” function on a desktop with the following specification: *Genuine Intel (R) CPU @ 2.00 GHz, 24 GB RAM, Windows 10, Python version 3.11.4*.

We report the mean time in seconds and standard deviation in milliseconds, the results are displayed in Table 4. Overall, the computational time of MrDcGene is comparable to DC-SIS, and slightly faster than SIS in $q = 1$ and $n<600$ scenarios. We can see the computational time for all three methods has a linear relationship with the predictor size *p*, which is expected since all three methods screen predictors one by one, as *p* increases, the computational time naturally increases as well. Interestingly, the number of observations *n* does not affect the computational time of SIS, Whether $n = 200$, 400, or 600, the computational time for SIS keeps relatively steady. This behavior may be attributed to the fact that SIS is based on Pearson correlation, and Python optimizes the calculation of this well-known correlation efficiently, so that it performs fast even for large *n*. On the other hand, the computational time for both DC-SIS and MrDcGene displays a linear relationship with $n^2$, which aligns with the statement that “the time complexity of DC-SIS is $O(n^2)$”^14^. Moreover, when $n = 600$, the computational time of DC-SIS and MrDcGene is already close to that of SIS. It is foreseeable that if *n* increases further, the computational time for DC-SIS and MrDcGene will exceed that of SIS. Regarding the response dimension *q*, it is noteworthy that it does not affect the computational time of DC-SIS and MrDcGene, this property is highly desirable as it implies that even when dealing with multi-omics data from various platforms, the computational time remains consistently efficient when employing MrDcGene for gene selection.

**Table 4:** Computational time for different *n*, *p*, and *q*.

		*p*	SIS			DC-SIS			MrDcGene		
			1000	2000	5000	1000	2000	5000	1000	2000	5000
$n = 200$	$q = 1$	Mean (s)	1.29	2.59	6.47	0.314	0.608	1.52	0.358	0.689	1.72
		sd (ms)	1.14	3.74	33.6	2.27	1.99	7.53	1.66	2.17	2.77
	$q = 3$	Mean (s)	NA	NA	NA	0.96	1.78	4.75	1.02	1.87	4.64
		sd (ms)	NA	NA	NA	98.6	18	106	121	16.6	58.9
	$q = 5$	Mean (s)	NA	NA	NA	0.99	1.94	4.97	1.17	2.02	5.05
		sd (ms)	NA	NA	NA	21	15.7	179	248	13.8	85.2
$n = 400$	$q = 1$	Mean (s)	1.32	2.64	6.62	0.606	1.22	3.04	0.661	1.33	3.32
		sd (ms)	12.5	28.7	15.3	0.67	15.3	13.4	1.24	14.9	12.2
	$q = 3$	Mean (s)	NA	NA	NA	3.49	7.28	17.8	3.62	7.27	18.3
		sd (ms)	NA	NA	NA	8.42	95.8	262	85.3	352	215
	$q = 5$	Mean (s)	NA	NA	NA	3.81	7.71	19.3	3.89	7.82	19.4
		sd (ms)	NA	NA	NA	33.6	101	630	49.7	183	486
$n = 600$	$q = 1$	Mean (s)	1.34	2.69	6.75	0.95	1.88	4.75	1.02	2.04	5.07
		sd (ms)	1.27	9.76	36.3	21.2	24	15.3	2.45	17.4	15.2
	$q = 3$	Mean (s)	NA	NA	NA	7.66	15.2	38.7	7.64	15	37.7
		sd (ms)	NA	NA	NA	207	277	643	243	189	726
	$q = 5$	Mean (s)	NA	NA	NA	8.2	16.9	42.1	8.22	16.5	41.3
		sd (ms)	NA	NA	NA	230	439	310	153	245	460

### Real data analyses

In this section we present the detailed results of the TCGA-LUAD data analysis. The data we used in this article was downloaded from TCGA official portal (https://portal.gdc.cancer.gov/) on April 13th, 2020, using TCGA-Assembler 2 package written in R^17,18^. Altogether we have data collected from five platforms: Gene Expression (GE), Reverse Phase Protein Array (RPPA), miRNA, Copy Number Variation (CNV), and Methylation (ME) for each of the 310 LUAD patients, interested readers can refer to the "Methods" section for detailed data pre-processing procedures performed prior to analyses. The modeling aim is to detect important associations between these platforms and the gene expression, and hence locate active genes influencing the LUAD disease.

Important associations fall broadly into at least one of two scenarios. Scenario 1: a predictor is associated with the expression of only a few gene components, but each component has a strong strength of association; and/or Scenario 2 (named pleiotropy in genetic terminology): a predictor is associated with the expression of several gene components, but each component may have a weak strength of association. To clearly distinguish between these two scenarios and to better interpret the results, we explore two different searching directions. Specifically, on one hand, we apply the maximum ratio criterion^19^ (see Methods, "Threshold selection") to all dependence scores and select the most important genes across all predictors and all subgroups (scenario 1). On the other hand, we again apply the maximum ratio criterion to each subgroup and select the most important genes across all predictors at each subgroup. Then, we summarize the maximum dependence scores and the total number of times each predictor is selected across all subgroups. The genes with the highest counts are then selected for scenario 2. In the following, we perform these steps separately for each of the four different studies below.

**Study 1**: Association between GE and RPPA-Protein.

**Figure 4:**
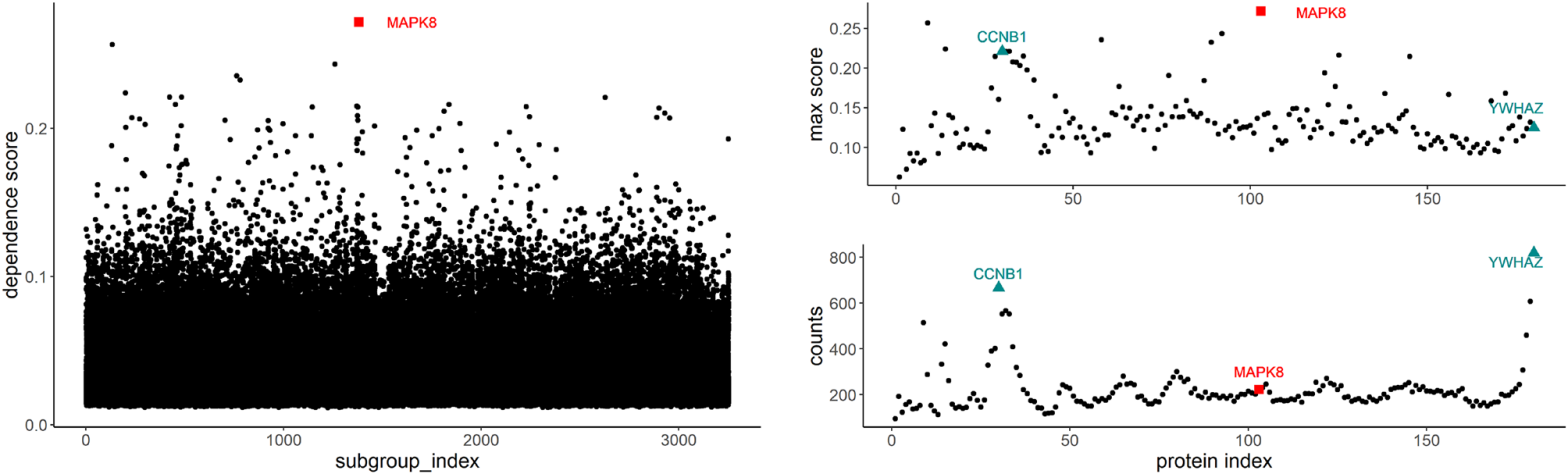
Left: The 585,360 dependence scores between gene expression and RPPA protein across all predictors and all subgroups; *MAPK8* is selected (in red square shape); Right: the maximum dependence score (top) and count of being selected (bottom) for each of the $p=180$ predictors across all of the 3252 subgroups; *CCNB1* and *YWHAZ* (in cyan triangular shape) are selected.

 For the RPPA-Protein platform data, the MrDcGene approach selects three important genes in total. They are *MAPK8* (in red square shape), *CCNB1*, and *YWHAZ* (in cyan triangular shape). Gene *MAPK8* has the highest dependence score among all of the $3252\times 180=585,360$ scores (left panel of Fig. 4). Judging from the right bottom panel of Fig. 4, its total count of being selected is relatively low, which represents an example of aforementioned scenario 1. This finding quantitatively confirms the reports in^20^, who claimed that *MAPK8* was one of eight key genes that were associated with Speckle-type POZ protein, which regulated LUAD cell response to radiation. Our second finding is *CCNB1* (the first cyan triangle on right panel of Fig. 4), from the top right panel of Fig. 4, the maximum score of *CCNB1* is relatively high, and from the bottom right panel of Fig. 4, it is also selected by approximately 700 subgroups, indicating a broad association. We can conclude that gene *CCNB1* falls into both scenario 1 and scenario 2, and it is important in associating with the gene expression of LUAD patients. This finding further confirm some other reports in the literature. For example, Liu et al. viewed *CCNB1* as a potential biomarker for diagnosis and prognosis target of LUAD^21^, and Li et al. reported that *CCNB1* was closely related with survival rate in LUAD^22^.

**Figure 5:**
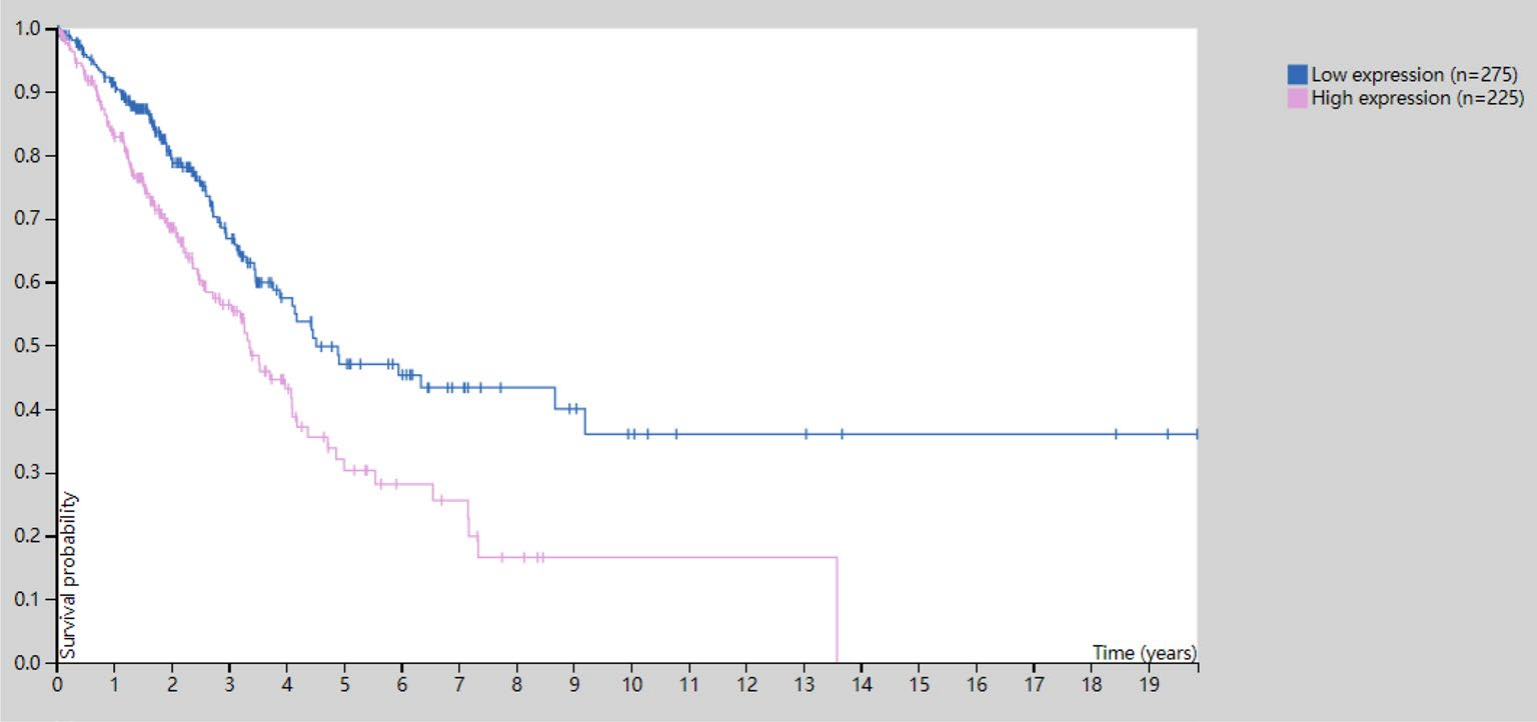
Kaplan-Meier plot of YWHAZ. Source: https://www.proteinatlas.org/ENSG00000164924-YWHAZ/pathology/lung+cancer/LUAD.

Our third finding, *YWHAZ* (the second cyan triangle on right panel of Fig.4), represents a classic example of Scenario 2, where its maximum score (top right panel of Fig. 4) shows no impressive marginal relationship with any subgroup of GE, however, its total count of selection is the highest (selected by over 800 subgroups, bottom right of Fig. 4). To the best of our knowledge, our report of *YWHAZ* in LUAD is a novel finding. We do not find any literature explicitly report its association with the LUAD disease, except in Ref.^23^ Gan et al. concluded that *YWHAZ* might be a potential biomarker of diagnosis, prognosis and chemoresistance in several cancers. In order to justify our new finding, we visually check that different expressions of *YWHAZ* affect the survival rate of LUAD patients differently using the Kaplan-Meier plot from The Human Protein Atlas (see Fig. 5).

**Study 2**: Association between GE and miRNA.

**Figure 6:**
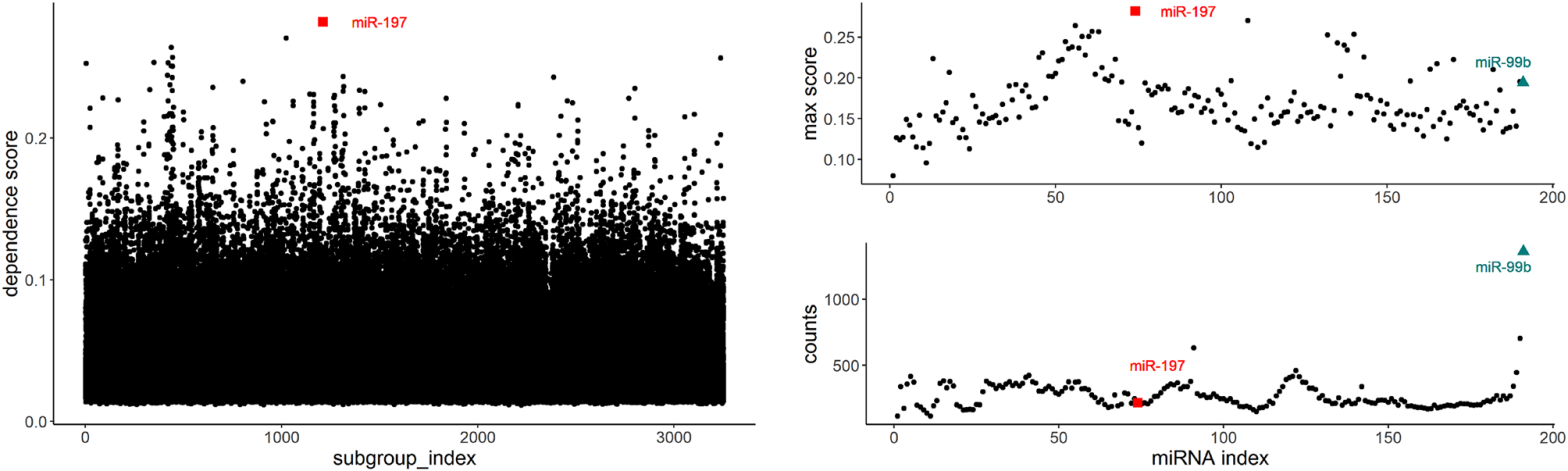
Left: The 621,132 dependence scores between gene expression and miRNA across all predictors and all subgroups; *miR-197* (in red square shape) is selected; Right: the maximum score (top) and count of being selected (bottom) for each of the $p=191$ predictor across all subgroups; *miR-99b* (in cyan triangular shape) is selected.

For the miRNA data, we select two important genes: *miR-197* (in red square shape) and *miR-99b* (in cyan triangular shape). The left panel of Fig. 6 demonstrates all of the $3252\times 191 = 621,132$ dependence scores, and the right panel of Fig. 6 summarizes the maximum dependence score (top) and also the total count of being selected (bottom) for each predictor across all of the 3252 subgroups for miRNA data. We can see that *miR-197*, locating from the 1211th subgroup, has the highest marginal dependence score. Our second finding, *miR-99b*, has a moderate level of importance score across all subgroups judging from the maximum score plot (top right of Fig. 6), however, its astonishing count make it shine because it is selected by over 1400 subgroups (bottom right of Fig. 6). These two findings confirm some other reports in the literature. For example, Chen and Yang claimed that *miR-197-3p* up-regulation was detected within LUAD tissues when comparing with adjacent noncancerous tissues, and they also confirmed that *miR-197-3p* directly targets the lysine 63 deubiquitinase (*CYLD*) gene, and the expression of *miR-197-3p* is negatively associated with *CYLD* expression within LUAD cell lines^24^. Kang et al. found that *miR-99b* was down-regulated in patients with lung cancer, noticed that the overexpression of *miR-99b* induced a reduction in *FGFR3* expression, and they suspected that *miR-99b* may be a potent tumor suppressor and a potential therapeutic tool for lung cancer patients^25^.

**Study 3**: Association between expressions of only five genes and integrated CNV and ME vector.

**Figure 7:**
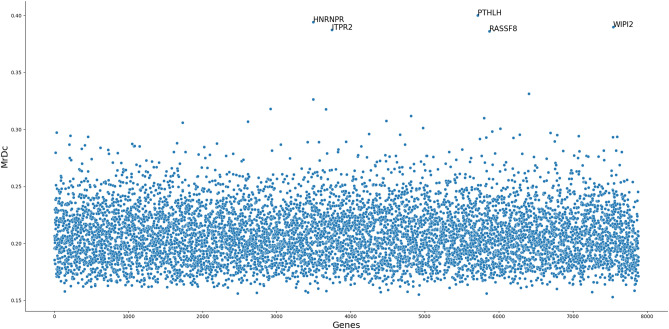
The 7, 885 dependence scores between gene expression of five specific genes and each of the 2-dimensional matched CNV and ME vector.

 In this study we only consider five specific genes that are known to be important for LUAD disease via the report from http://www.cancerindex.org/geneweb/X1501.htm. Specifically, the response vector are expressions of five genes, i.e., *EGFR, KRAS, TP53, MKI67,* and *ALK* (i.e., $n\times q = 310 \times 5$). Then we match CNV and ME together as an entire unit through their common genes ($n\times p\times d = 310 \times 7,885\times 2$). The dependence scores of the 7, 885 two-dimensional predictor vectors are demonstrated in Fig. 7. The maximum ratio criterion selects five genes as important findings and they are: *HNRNPR, ITPR2, PTHLH, RASSF8* and *WIPI2*, which, as can be visually observed, far outweigh the rest of the genes. Some of our findings confirm other reports in the literature, for example, Wei et al. noticed up-regulation of *RASSF8* in LUAD patients^26^; kucherlapati found that *WIPI2* is over expressed in *EGFR* activated tumors^27^; Manenti et al. noticed the polymorphisms in *KRAS* and *PTHLH* loci were associated with tumor prognosis^28^. Note that the five genes we chose as response components to consider their gene expressions are different from the five genes that are selected by the proposed MrDcGene approach from the CNV and ME vector. This may indicate interactions of these ten genes in influencing LUAD.

**Study 4**: Association between expressions and integrated CNV and ME vector for all matched genes.

 As an extension of the Study 3, we now let the data speak and consider all 7885 gene expressions. Since these three platforms (GE, CNV and DNA methylation) represent different functions of the same gene, we are able to integrate the three platforms by their matching genes. After the gene matching process, each predictor is a $310\times 7885\times 2$ array (CNV for the 1st dimension and DNA methylation for the 2nd dimension). The response is a $310\times 7885$ matrix (GE).

**Figure 8:**
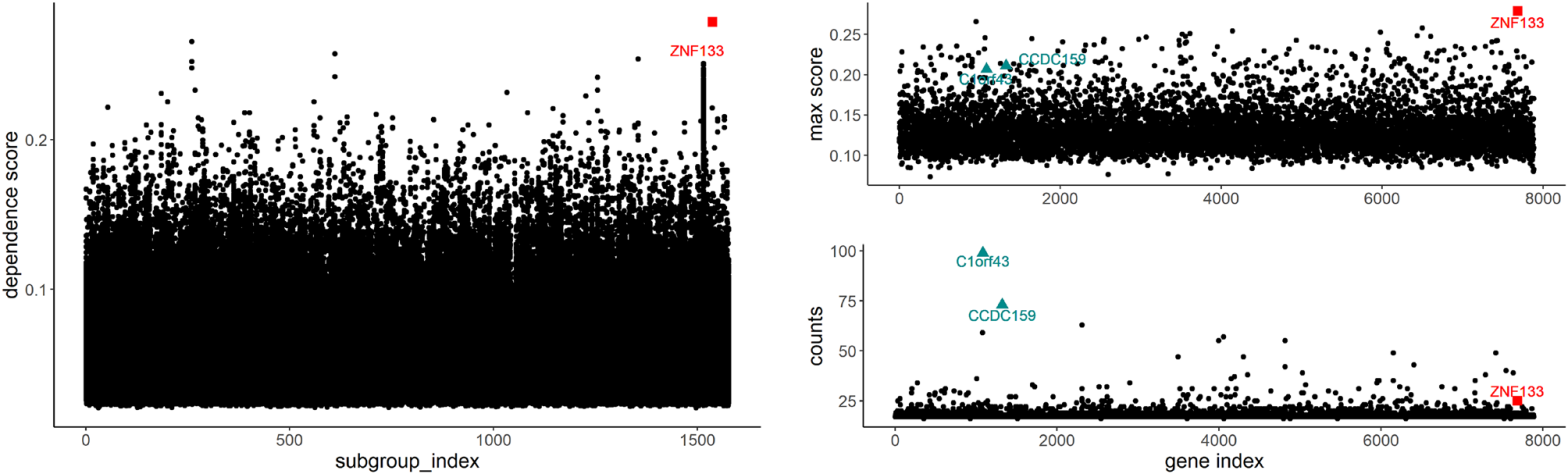
Left: The 12,434,645 dependence scores between gene expression and integrated GE and CNV of all matched genes across all predictors and all subgroups; *ZNF133* (in red square shape) is selected; Right: the maximum score (top) and count of being selected (bottom) for each of the $p=191$ predictor across all subgroups; *C1orf43* and *CCDC159* (in cyan triangular shape) are selected.

**Figure 9:**
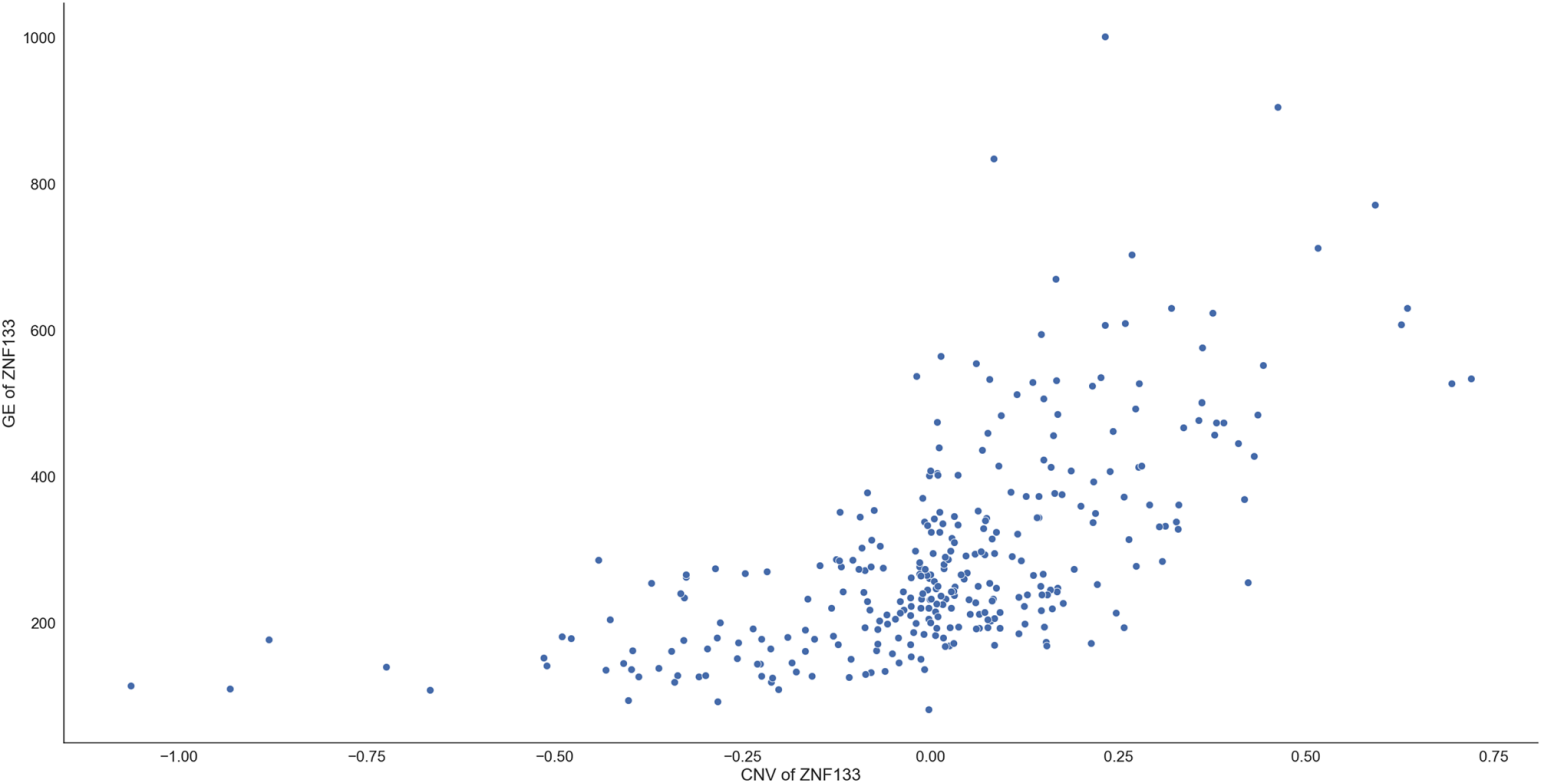
Scatter plot of GE versus CNV for the gene ZNF133.

To solve the weak signal-to-noise ratio problems existing in the response vector as demonstrated in the simulation section, we again divide the response into 1577 ($=7885/5$) subgroups with each subgroup having five gene expressions. After applying a max-ratio criterion to the entire $7885\times 1577=12,434,645$ dependence scores (see the left panel of Fig. 8), we select gene *ZNF133* from the 1537th subgroup (in red square shape), which is an example of Scenario 1. The scatter plot shows a surprisingly nonlinear relationship between GE and CNV of *ZNF133* (see Fig. 9), which would be a problem if a linear or inappropriate parametric models were used. Applying the max-ratio criterion on each subgroup and then summarizing the maximum score and count of each predictor vector across all subgroups, we identify another two influential genes, *C1orf43* and *CCDC159* (marked as triangular cyan shapes in the right panel of Fig. 8). Our finding of *C1orf43* quantitatively confirm some other reports of the literature. For example, Zheng et al. identified and validated *C1orf43* as one of the optimal diagnostic and prognostic signature for overall survival in LUAD^29^. To our best knowledge, *ZNF133* and *CCDC159* have not been found in literature yet.

## Discussion

More than 800 genes have been identified to be associated with prognosis and disease progression in LUAD. However, existing studies have focused on single outcomes, such as survival rates, clinical endpoints or binary affective statuses. Single-omics analysis has several limitations and therefore cannot decipher the complex physiological characters of cancer. As a breakthrough, multi-omics data have attracted a lot of attention in cancer research^5,7^. However, most multi-omics data analyses still follow a “phenotype-first” structure, where clinical endpoints such as survival or tumor measurements are used as response variables and genes are sought through associations between these endpoints and a superimposed long vector containing all multi-omics platforms. These methods are only useful when the disease has a simple structure. For complex diseases like cancer progression, finding phenotypic associations may not be as straightforward as exploring intracellular pathogenic mechanisms. Responding to this need, the MrDcGene selection procedure we present in this article does not restrict who is response or phenotype; instead, it measures associations between multiple platforms simultaneously when both sides can be high-dimensional.

Applying the MrDcGene approach to LUAD multi-omics data, we identify several important genes as potential biomarkers for lung cancer. Specifically, *MAPK8*, *CCNB1*, and *YWHAZ* are found from the RPPA-protein platform (Study 1); *miR-197* and *miR-99b* from the miRNA platform (Study 2); *HNRNPR*, *ITPR2*, *PTHLH*, *RASSF8* and *WIPI2* from the the integrated CNV and ME for the five specific genes (Study 3); and *ZNF133*, *C1orf43*, and *CCDC159* from the integrated CNV and ME for the entire expressions of 7885 genes (Study 4). Some of these findings, *MAPK8, CCNB1, C1orf43*, quantitatively confirm reports in the literature using very different methods, foci, and studies^20,21,29^. In addition, we also identify several novel scientific findings that have not been reported in the literature yet, such as *ZNF133, CCDC159, YWHAZ, HNRNPR, ITPR2, PTHLH*, and *WIPI2*, regarding their important role in relation to LUAD.

It is worth pointing out that the subgrouping procedure is able to differentiate two types of association in the multi-omic data. Scenario 1: a predictor is associated with the expression of only a few gene components, but each component has a strong strength of association; and Scenario 2 (named pleiotropy in genetic terminology): a predictor is associated with the expression of several gene components, but each component may have a weak strength of association. We detect *ZNF133* as an example of scenario 1 and *CCDC159* as an example of scenario 2 as two novel findings. It is demonstrated through the Simulation and Study 4 that the MrDcGene approach performs well in the complex structure when both sides are in high dimension and signal-to-noise ratio is very weak. In addition, it is ideal for multiple platforms with no extra computational cost.

## Methods

### Multivariate rank

In this section, we introduce the main scheme of the MrDcGene approach. For more detailed techniques, we refer the readers to Zhao and Fu^14^. Let $\mathbb {R}^d$ denote the original *d*-dimensional space, and $[0,1]^d$ denote the *d*-dimensional unit hypercube. Let $\mathcal {P}(\mathbb {R}^d)$ denote the families of all probability distributions on $\mathbb {R}^d$, and $\mathcal {P}_{ac}(\mathbb {R}^d)$ denote the families of Lebesgue absolutely continuous probability measures on $\mathbb {R}^d$. Let $\mathcal {U}^d$ denote the uniform distribution on $[0,1]^d$, and $C_n$ stands for the set of all permutations of $\{1, 2, \dots , n\}$. We use $\Vert \cdot \Vert$ to represent $\ell _2$ norm and $\langle \cdot \rangle$ to represent inner product.

If $\{X^1,X^2,\dots .X^n\}$ are samples of a univariate random variable *X* (i.e., the dimension $d=1$), it is easy to get the empirical rank by simply sorting $\{X^i\}_{i=1}^n$ and assigning $\{\frac{i}{n}\}_{i=1}^n$ to the sorted points. Then $\{\frac{i}{n}\}_{i=1}^n$ is a good discrete approximation of $\mathcal {U}^1$. If $\{\textbf{X}^i\}_{i=1}^n$ are samples of a multivariate vector with higher dimensions ($d>1$), it is not straightforward to find a multivariate rank to represent this sequence. Deb and Sen initiated a distribution-free and model-free dependence measure to construct multivariate rank and map the original data to a unit hypercube^30^.

The key idea of multivariate rank is to firstly generate a good discrete sequence as an approximation of $\mathcal {U}^d$ where $d>1$, and then allocate $\{\textbf{X}^i\}_{i=1}^n$ to such a discrete sequence. We adopt the low-discrepancy sequence, also known as the quasi-Monte Carlo (QMC) sequence, as our discrete sequence. Low-discrepancy sequences initially emerged in approximating the integral of functions on $[0, 1]^d$. To get a better approximation, people want to divide $[0, 1]^d$ more evenly, and use function values at these grid points to approximate the function on $[0, 1]^d$. The Monte-Carlo (MC) method generates a random sample from $\mathcal {U}^d$, however, the generated sequence may not be truly “evenly” distributed on $[0, 1]^d$ and the convergence rate is always $O(1/\sqrt{n})$^31^. Mathematicians came up with the quasi-Monte Carlo to achieve a faster convergence rate than the MC methods, by constructing a fixed-points set with low “discrepancy” with a convergence rate of order $O[(\log n)^d/n]$ or even $O[(\log n)^d/n^2]$^31^. Among these low-discrepancy sequences, Halton sequence^32^, Sobol’ sequence^33^ and Niederreiter sequence (also known as (t,m,s)-nets;^34^) have been thoroughly studied and their extensions (scrambled, truncated, etc) are widely applied in Finance and other fields. Current software like R or Python can easily generate such sequences with arbitrary dimensions.

The low-discrepancy sequences have multiple advantages than the random sequences in $\mathcal {U}^d$. Firstly, low-discrepancy sequences have the property of “low-discrepancy”. This means that the proportion of points in the sequence that fall into an arbitrary set *B* is close to the proportion of the measure of *B*. If we have the same number of points in both sequences, the low-discrepancy sequences may be more evenly distributed on $[0,1]^d$ than random sequences. Secondly, low-discrepancy sequences have better convergence rate, and one doesn’t need too many points to get a good approximation of $\mathcal {U}^d$, which is particularly agreeable for high dimensional dataset. Thirdly, low-discrepancy sequences can be easily extended for extra samples. For example, if one extra observation is added, generating low-discrepancy sequences simply requires to add one more point without the need of recalculating the entire sequence, which greatly save computational cost. Fourthly, low-discrepancy sequences are fixed sequences and it leads to reliable and reproducible consequence. If one generates a low-discrepancy sequence multiple times, exactly the same results are achieved every time on every machine. However, it may not be the case if one generates random sequences multiple times.

There have been a number of studies to improve the performance of low-discrepancy sequences in high dimensionality. Paskov and Traub demonstrated that QMC were still superior to MC sequences for high-dimensional integrals with 360-dimension^35^; Sobol et al. explored the Sobol’ sequence up to 16,384-dimension^36^; Joe and Kuo constructed Sobol’ sequences for 21,201-dimension data, showing that no weird patterns or collinearity issues occurred if the 21,201-dimension data that they generated were projected into any two-dimensional plots^37^.

Let $\mathcal {D}_n^{\textbf{X}}=\{ \textbf{X}^1,\textbf{X}^2,\dots ,\textbf{X}^n \}$ be the observed data, where *n* is the sample size. Let $\mathcal {H}_n^d:=\{\varvec{h}_1^d,\varvec{h}_2^d,\dots ,\varvec{h}_n^d\}$ denote a sample of a multivariate rank vector (we use the Sobol’ sequence for $d> 1$; and use $\{\frac{i}{n}\}_{i = 1}^n$ for $d = 1$). Let $\mu _n^{\textbf{X}}:=\frac{1}{n}\sum _{i = 1}^{n}\delta _{\textbf{X}^i}$ and $\nu _n:=\frac{1}{n}\sum _{i = 1}^{n}\delta _{h_i^d}$ be the empirical distributions on $\mathcal {D}_n^{\textbf{X}}$ and $\mathcal {H}_n^d$ respectively, where $\delta$ represents the Dirac measure. Then the empirical rank is defined as the optimal transportation map as

$$\begin{aligned} \hat{R}_n = \mathop {\text{argmin}}\limits _F\int \left\Vert \textbf{X}-F(\textbf{X})\right\Vert ^2 d\mu _n^{\textbf{X}}(\textbf{X})\ \text {subject to}\ F\#\mu _n^{\textbf{X}} = \nu _n, \end{aligned}$$

where $F\#\mu _n^{\textbf{X}} = \nu _n$ means *F* transports the distribution from $\mu$ to $\nu$, i.e., $F(\textbf{X})\sim \nu$ where $\textbf{X}\sim \mu$^38^. And it is equivalent to

$$\begin{aligned} \hat{\sigma }_n:=\mathop {\text{argmin}}\limits _{\sigma \in C_n}\sum _{i = 1}^{n}\left\Vert \textbf{X}^i-\varvec{h}_{\sigma (i)}^d\right\Vert ^2 = \mathop {\text{argmax}}\limits _{\sigma \in C_n}\sum _{i = 1}^{n}\left\langle \textbf{X}^i,\varvec{h}_{\sigma (i)}^d\right\rangle . \end{aligned}$$

Finally, the empirical rank is obtained as

1 $$\begin{aligned} \hat{R}_n(\textbf{X}^i) = \varvec{h}_{\hat{\sigma }_n(i)}^d,\ \forall ~i = 1,2,\dots ,n. \end{aligned}$$

This combinatorial optimization problem can be solved using modified Hungarian algorithm^39,40^. We can see that this multivariate rank transportation guarantees that the original data $\textbf{X}^i$ has been transported to $\hat{R}_n(\textbf{X}^i)$, which always lies in $[0,1]^d$, regardless of the original range of $\textbf{X}$.

### Multivariate rank distance correlation

Let $\textbf{X}$ and $\textbf{Y}$ be two multivariate vectors with dimensions $d_1$ and $d_2$, respectively. The optimal rank map $R^\textbf{X}(\textbf{X})$ transforms $\textbf{X}$ into $\mathcal {U}^{d_1}$, and the optimal rank map $R^{\textbf{Y}}(\textbf{Y})$ transforms $\textbf{Y}$ into $\mathcal {U}^{d_2}$. Then the multivariate rank distance covariance (MrDcov) of $\textbf{X}$ and $\textbf{Y}$ can be estimated as follows^14,41^

$$\begin{aligned} \widehat{MrDcov}^2(\textbf{X},\textbf{Y}) = \widehat{MrS}_1 +\widehat{MrS}_2-2\widehat{MrS}_3, \end{aligned}$$

where

$$\begin{aligned} \begin{aligned} \widehat{MrS}_1&= \frac{1}{n^2}\sum _{i=1}^{n}\sum _{j=1}^{n}\left\Vert \hat{R}_n^{\textbf{X}}(\textbf{X}^i) -\hat{R}_n^{\textbf{X}}(\textbf{X}^j)\right\Vert _{d_1}\left\Vert \hat{R}_n^{\textbf{Y}}(\textbf{Y}^i) -\hat{R}_n^{\textbf{Y}}(\textbf{Y}^j)\right\Vert _{d_2}, \\ \widehat{MrS}_2&= \frac{1}{n^2}\sum _{i=1}^{n}\sum _{j=1}^{n}\left\Vert \hat{R}_n^{\textbf{X}}(\textbf{X}^i)-\hat{R}_n^{\textbf{X}}(\textbf{X}^j)\right\Vert _{d_1} \frac{1}{n^2}\sum _{i=1}^{n}\sum _{j=1}^{n}\left\Vert \hat{R}_n^{\textbf{Y}}(\textbf{Y}^i)-\hat{R}_n^{\textbf{Y}}(\textbf{Y}^j)\right\Vert _{d_2},\\ \widehat{MrS}_3&= \frac{1}{n^3}\sum _{i=1}^{n}\sum _{j=1}^{n}\sum _{l=1}^{n}\left\Vert \hat{R}_n^{\textbf{X}}(\textbf{X}^i)-\hat{R}_n^{\textbf{X}}(\textbf{X}^l)\right\Vert _{d_1} \left\Vert \hat{R}_n^{\textbf{Y}}(\textbf{Y}^j)-\hat{R}_n^{\textbf{Y}}(\textbf{Y}^l)\right\Vert _{d_2}. \end{aligned} \end{aligned}$$

Accordingly, the multivariate rank distance correlation (MrDc) can be estimated as

$$\begin{aligned} \widehat{MrDc}(\textbf{X},\textbf{Y}) =\frac{\widehat{MrDcov}(\textbf{X},\textbf{Y})}{\sqrt{\widehat{MrDcov}(\textbf{X},\textbf{X}) \widehat{MrDcov}(\textbf{Y},\textbf{Y})}}. \end{aligned}$$

Finally, we use the MrDc as a criterion for gene selection. That is, we compute a dependnece score for each of the predictor,

2 $$\begin{aligned} \hat{\omega }_j:=\widehat{MrDc}(\textbf{X}_j,\textbf{Y}), j = 1,2,\dots ,p, \end{aligned}$$

to measure the association strength between $\textbf{X}_j, j = 1,2,\dots ,p$ and the response $\textbf{Y}$. Here, both $\textbf{Y}$ and $\textbf{X}_j$ can be high-dimensional, as multi-omics data are needed. Note that no model structure or distribution assumptions are involved in the Methodology, hence, it ensures the model-free and distribution-free nature of the MrDcGene procedure. Furthermore, it does not matter who is response vector and who is predictor vector, which further increases the flexibility and application scope of the MrDcGene procedure in multi-omics data analysis. For convenience, $\textbf{Y}$ is still referred to as the response (gene expression) and $\textbf{X}_j$ (CNV, ME, miRNA, RPPA-Protein) as the predictor in this article.

### Threshold selection

The MrDcGene does not provide p-values or significance tests, which is a common problem for variable selections in general. Therefore, we need to pre-determine a threshold so that a subset of important predictors can be selected. After computing the $\hat{\omega }_j$ scores from the Equation (2), all predictors can be ranked from the most important to the least important as $\hat{\omega }_{(1)}\geqslant \hat{\omega }_{(2)}\geqslant \cdots \geqslant \hat{\omega }_{(p)}$. We follow the max-ratio criterion proposed in^19^ to determine such a threshold. Based on the idea that a noise predictor likely has a score close to zero, we expect to see a large $\hat{s}_0$ value at the separation point, where $\hat{s}_0$ is defined as

3 $$\begin{aligned} \hat{s}_0 = \mathop {\text{argmax}}\limits _{1\leqslant j\leqslant p-1}~\hat{\omega }_{(j)}/\hat{\omega }_{(j+1)}. \end{aligned}$$

### Algorithm of MrDcGene

The scheme of the entire gene selection process for the multi-omics data is described as follows. Denote *p* as the number of predictors, *d* as the number of platforms, and *q* as the number of gene expression components.

**Algorithm 1:**
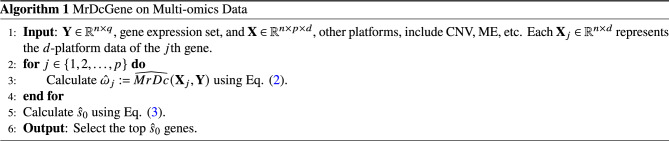
MrDcGene on Multi-omics Data.

### Real data pre-processing

The raw data we used in this article was downloaded from TCGA official portal (https://portal.gdc.cancer.gov/) on April 13th, 2020, using TCGA-Assembler 2 package written in R^17,18^. Altogether we have data collected from five platforms: GE, RPPA, miRNA, CNV, and ME for each of the 310 LUAD patients. Before applying MrDcGene, we pre-process the data as follows:

- *Gene Expression*. We download the normalized *RNAseq* gene data. Following the same routine as in^42^, RNA with expression value (normalized read counts) $>1$ in approximately 70% samples are retained. The GE data is arranged in a $310\times 16,260$ matrix. This is the response data.
- *Reverse Phase Protein Array Data-protein*. The protein data is collected from RPPA. This is a relatively small data set. We only drop probes with NA values. The RPPA is arranged in a $310\times 180$ matrix.
- *miRNA*. We download the *hg19.mirbase20_RPM* data, where miRNA are mapped to the human genome reference sequence “hg19” and mirbase20, and normalized as reads per million (RPM) value. Following the same routines in^43^ and^42^, we filter out the low-abundance miRNA, and keep miRNA with expression value $>10$ in 80% samples. The miRNA data is arranged in a $310\times 191$ matrix.
- *DNA methylation*. We download $methylation\_450k$ data, which is an advanced and comprehensive version compared with its predecessor, $methylation\_ 27k$. Following the routine in^44^, we remove zero entries, sort all the values, and obtain the overall 25% quantile. Then we drop the genes whose maximum values are less than the overall 25% quantile. The ME is arranged in a $310\times 14,676$ matrix.
- *Copy number variation*. We download the *nocnv.hg19* data, which eliminates some germline CNV. We remove many duplicated variables that have different gene names but actually identical CNV values for all patients. The CNV data is arranged in a $310\times 7885$ matrix.

**Figure 10:**
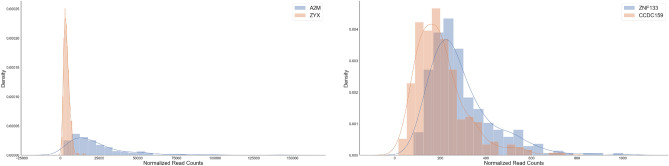
The histogram plots of gene expression data for genes A2M and ZYX (left panel) and genes ZNF133 and CCDC159 (right panel), as four examples.

After exploring the expression values of several individual genes, we noticed some unusual challenges such as extremely long tails, heavy outliers, very different ranges existing in the same dataset. For example, the left panel of Fig. 10 demonstrates that gene ZYX has an expression with high peak but narrow range (from 0 to 10, 000), while the expression of the gene A2M has a much flatter peak with an extremely wide range extending from − 25,000 to 150,000. The right panel of Fig. 10 illustrates the expression of genes ZNF133 and CCDC159, showing severe right skew and heavy tails, likely violating the normal distribution assumption. These challenges may trap many gene selection methods, but MrDcGene performs well in this case as a model-free and distribution-free method (see Ref.^14^ for more detailed simulation demonstrations).

### Gene selection procedure for multiple platforms

As illustrated in the simulation section, it is feasible for the MrDcGene approach to integrate multiple platforms as one unit (i.e., integrating all four platforms, RPPA, miRNA, DNA methylation, and CNV, into a single four-dimensional vector). However, application-wise, these four platforms do not have a common component that can be used to integrate them, and therefore it does not make biological sense to unite all four platforms. Instead, we integrate DNA methylation and CNV into a bivariate dimensional vector because these two platforms can be paired by common matching genes; and we model RPPA and miRNA individually. In addition, modeling the 16,260-dimensional response vector as one unit would mislead subsequent selection process if most of the gene expression components were actually irrelevant. Following the grouping procedure that is introduced in the simulation section, we divide the 16,260-dimensional gene expression data into 3252 subgroups, each of which includes five genes. We then compute the marginal dependence score for each predictor in each subgroup.

## Data Availability

The raw datasets are publicly available from TCGA official portal (https://portal.gdc.cancer.gov/), the datasets used and/or analysed during the current study are available from the corresponding author on reasonable request.
